# The Effect of a Physical Activity Program on the Total Number of Primary Care Visits in Inactive Patients: A 15-Month Randomized Controlled Trial

**DOI:** 10.1371/journal.pone.0066392

**Published:** 2013-06-21

**Authors:** Maria Giné-Garriga, Carme Martin-Borràs, Anna Puig-Ribera, Carlos Martín-Cantera, Mercè Solà, Antonio Cuesta-Vargas, on behalf of the PPAF Group

**Affiliations:** 1 Department of Physical Activity and Sport Sciences, FPCEE Blanquerna, Universitat Ramon Llull, Barcelona, Spain; 2 Department of Physical Therapy, FCS Blanquerna, Universitat Ramon Llull, Barcelona, Spain; 3 Department of Physical Activity and Sport Sciences, Universitat de Vic, Vic, Spain; 4 Research Unit of Barcelona, Primary Healthcare Research Institution IDIAP Jordi Gol, Barcelona, Spain; 5 Department of Medicine, Universitat Autònoma de Barcelona, Barcelona, Spain; 6 Department of Physical Therapy, Universidad de Málaga, Málaga, Spain; Universidad Europea de Madrid, Spain

## Abstract

**Background:**

Effective promotion of exercise could result in substantial savings in healthcare cost expenses in terms of direct medical costs, such as the number of medical appointments. However, this is hampered by our limited knowledge of how to achieve sustained increases in physical activity.

**Objectives:**

To assess the effectiveness of a Primary Health Care (PHC) based physical activity program in reducing the total number of visits to the healthcare center among inactive patients, over a 15-month period.

**Research Design:**

Randomized controlled trial.

**Subjects:**

Three hundred and sixty-two (n = 362) inactive patients suffering from at least one chronic condition were included. One hundred and eighty-three patients (n = 183; mean (SD); 68.3 (8.8) years; 118 women) were randomly allocated to the physical activity program (IG). One hundred and seventy-nine patients (n = 179; 67.2 (9.1) years; 106 women) were allocated to the control group (CG). The IG went through a three-month standardized physical activity program led by physical activity specialists and linked to community resources.

**Measures:**

The total number of medical appointments to the PHC, during twelve months before and after the program, was registered. Self-reported health status (SF-12 version 2) was assessed at baseline (month 0), at the end of the intervention (month 3), and at 12 months follow-up after the end of the intervention (month 15).

**Results:**

The IG had a significantly reduced number of visits during the 12 months after the intervention: 14.8 (8.5). The CG remained about the same: 18.2 (11.1) (*P* = .002).

**Conclusions:**

Our findings indicate that a 3-month physical activity program linked to community resources is a short-duration, effective and sustainable intervention in inactive patients to decrease rates of PHC visits.

**Trial Registration:**

ClinicalTrials.gov NCT00714831

## Introduction

Primary care physicians are the major health care providers for people with multiple morbidities [1], [2]. In Britain, people with chronic health problems account for about 80% of consultations in primary care, and people with three or more chronic problems are over four times as likely to see their general practitioner (GP) compared to those who reported no conditions [3]. The rates are similar in Spain, with 67% multiple morbidity in the group of individuals 65 years of age or older [2]. Multiple morbidity has been shown to be associated with poor functional status [4], lower quality of life [5], an overloaded care system, especially at the primary care level [6], and a greater use of specialized care [1]. There is evidence to suggest that inactive individuals, having a greater risk of having multiple chronic diseases, are over-using the resources of primary care centers and increasing consultation rates of the health services [2]. In England, physical inactivity is estimated to cost the economy around 8.3 billion pounds annually, of which between 1 and 1.8 billion pounds is associated with the treatment of physical inactivity related diseases [7]. In Europe, inactive people with multiple morbidities contribute to an increased demand for medical and social care, and are associated with increased health costs [8].

The health benefits of exercise are probably the most important self-help treatment available [9]. Despite the health benefits of regular exercise, the Spanish population is mainly inactive [10], [11]. Savings due to increased physical activity in the population have been shown for different countries (e.g. Switzerland, Austria, and USA) [12]. Effective promotion of exercise could result in substantial healthcare cost savings in terms of direct medical costs, such as the number of consultations and medication, but this is hampered by our limited knowledge of how to achieve sustained increases in physical activity [13].

It has been documented that there are problems with the quality and continuity of care provided to older patients, including failure to refer to appropriate community services [14], [15], [16]. In a universal health care system, the government pays for almost all health care costs. Thus, most aspects of the health care system such as hospitals, primary care centers and prescription drugs, are controlled by the government. Like other nations with a universal health care system (e.g. Germany, Denmark and Sweden), Spain has had to deal with the problem of ever-growing health care expenses, causing a strain on government budgets and tax revenue increases. The search for solutions to increasing rates of primary care use and the resulting overuse has focused attention on reducing the demand for primary care services; the inactive adult population is a natural target for these efforts.

In view of the above problems, it is of interest to determine whether physical activity interventions affect the rates of primary care use. To date, there is limited evidence on the cost-effectiveness of primary care based physical activity programs. Previous reviews of physical activity programs have assessed their effects on health and functional outcomes, as well as on other types of service use [9], [17]. None of them, to our knowledge, have examined their effects on primary care use in terms of the number of consultations.

Thus, this randomized controlled trial was conducted to assess the effectiveness of a primary care based physical activity program linked to community resources on reducing the total number of consultations to the healthcare center. We also assessed the effectiveness of the program on the health-related quality of life of the patients.

## Methods

### Study Design

The protocol for this trial and supporting CONSORT checklist are available as supporting information; see Checklist S1 and Protocol S1. A 2-arm randomized controlled trial was conducted comparing the effectiveness of a 12-week physical activity intervention linked to municipal resources, and usual care combined with social education meetings. Study design details are described elsewhere [18].

### Ethics Statement

Written informed consent was obtained from all subjects prior to participation, and the Clinical Research Ethics Committee of the Research Institute in Primary Care Jordi Gol gave approval for the study. The study was performed in accordance with the declaration of Helsinki II.

### Participants

Participants were recruited from eight primary health care centers (PHC) in the Barcelona area and surroundings from 63 randomly selected PHC in Catalonia. Eligibility criteria included patients aged 18 to 85, with at least one chronic disease (diabetes mellitus, COPD and asthma, hypercholesterolemia, hypertension, chronic heart failure, obesity, osteoarticular chronic problems, and chronic muscular-skeletal pain), independent in rising from a chair and walking with or without a technical aid, who were physically inactive, as determined by the following question screening tool: “As a rule, do you do at least half an hour of moderate or vigorous exercise (such as walking, cycling or a sport) on five or more days of the week?” [19].

Individuals were ineligible for the study if they were unable to walk, were undergoing an exercise program, had a diagnosis of severe dementia (not able to understand and/or follow verbal commands), or had had a stroke, hip fracture, myocardial infarction or had undergone hip- or knee- replacement surgery within the previous 6 months.

### Sample Size Calculation

Sample size calculation was estimated for significant changes in total number of visits to the primary healthcare centers. Three hundred and forty-two participants (171 per group) were needed to detect a 15% decrease in the number of visits 12 months after the end of the intervention, with a power of 80% and an α = 0.05, a standard deviation of 30% of the mean, and a 20% dropout rate. Three hundred and sixty-two participants were recruited for the study.

### Recruitment and Randomization Procedures

The recruitment process took place in 8 PHC during the first three months of 2009. During April-August 2008, 63 randomly selected PHC in Catalonia were informed and the trial was presented to the 54 centers that showed interest in participating. Of these, the first eight centers which volunteered to participate underwent the trial. Two health professionals, who were selected on a voluntary basis from each of the participating centers, were trained over the study protocol and subjects selection, and were blinded to the study group assignment of their patients. During the recruitment period, the opportunity to participate in the study was offered daily to all patients, who by systematic random sampling were previously identified in the lists of the two health professionals. Patients who met the inclusion criteria and agreed to participate were further contacted for an interview with a researcher, duly informed about the study, and signed the informed consent.

Those found eligible were administered a baseline questionnaire with demographic data. Afterwards, they were randomly allocated to the intervention (IG) or control group (CG), using a centrally generated variable-sized block design. One hundred and eighty-three patients (n = 183; 68.3 (8.8) years; 118 women) were randomly allocated to the physical activity program (IG). One hundred and seventy-nine patients (n = 179; 67.2 (9.1) years; 106 women) were allocated to the control group (CG).

**Figure 1:**
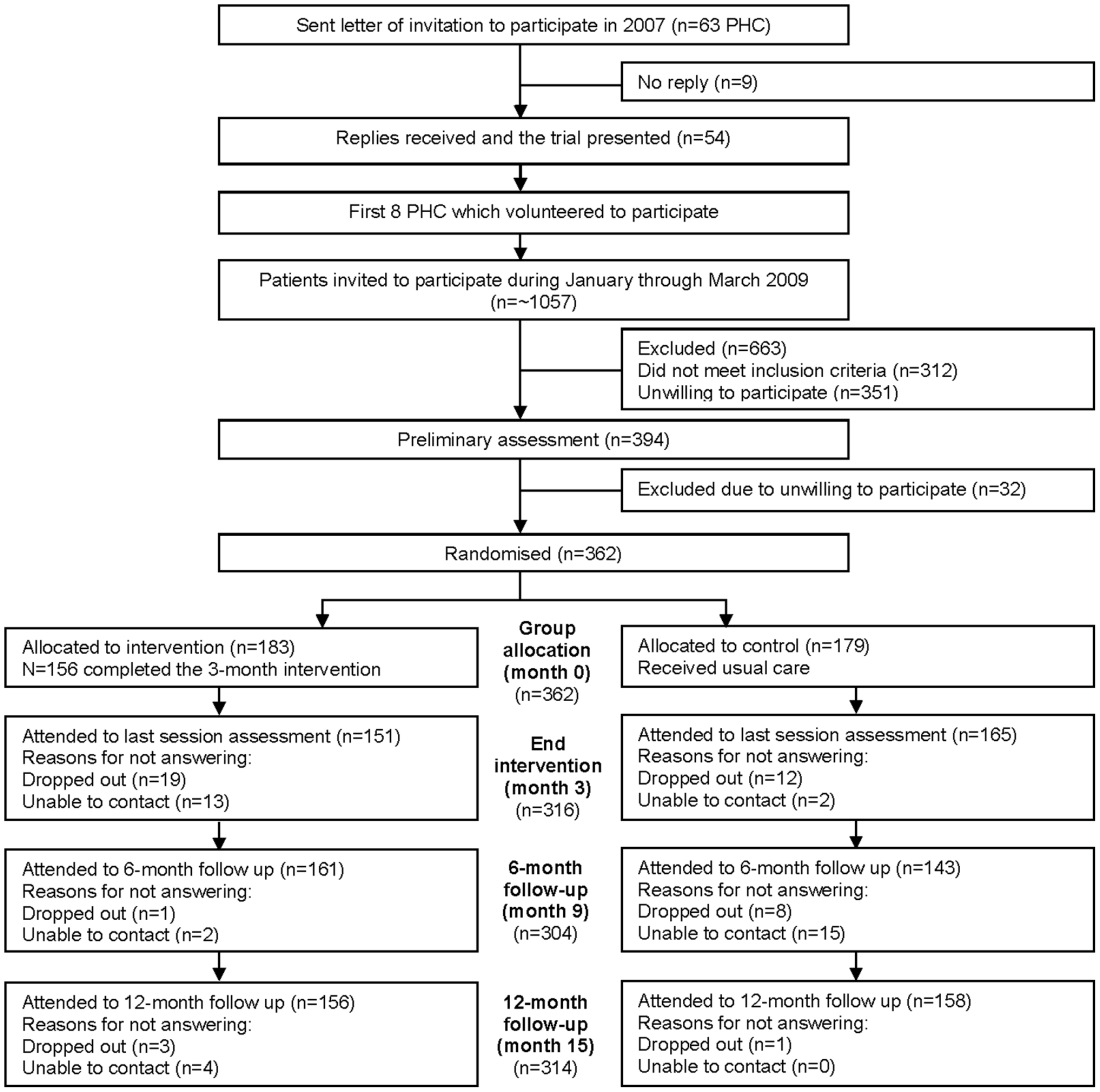
Flowchart of participant’s recruitment and trial design. *Note:* During follow-ups of both the intervention and the control groups, some participants were contacted by phone, increasing the number of attendees from previous follow-up.

The study personnel involved in the recruitment process and randomization log were not involved in screening, testing, or training procedures. Figure 1 shows the flowchart of participants through the study following the Consolidated Standard of Reporting Trials (CONSORT) flow diagram [20].

### Outcome Measures

Demographic and health data was collected at baseline (month 0) (age, gender, weight, height, body mass index, marital status, current medication, associated pathologies, and the number of metabolic equivalents (MET level x minutes of activity x events per week) documented with the short-version International Physical Activity Questionnaire [21].

The primary outcome measure was the total number of consultations to the healthcare centre, with the total number of visits during the twelve months prior (month 0) and after the program (month 15) being recorded. Differing from the protocol, we decided to collect the total number of visits during the twelve months prior and after the program rather than the six months in order to avoid any bias. The outcome measure related to physical activity levels is not shown in the present article. Consultations included face-to-face and home visits by GPs or nurses, and also out-of-hours visits to the healthcare centre according to three possible modalities: (a) appointment demanded by the patient, (b) planned visit to either the GP or nurse, and (c) an emergency visit. Telephone calls or hospital visits were not included as reliable data was unavailable. Three assistant researchers, blinded to the group allocation, obtained the primary outcome measure from the computerized clinical records.

Secondary outcomes were: (a) self-reported physical function, (b) physical composite score, and (c) mental composite score, documented via the SF-12 version 2 survey [22], and calculated using norm-based scoring algorithms [23]. The SF-12 questionnaire has been validated in Spanish [24] showing a high level of internal consistency (alpha = 0.71 to 0.99) and test-retest reliability (ICC = 0.58 to 0.99).

Self-reported outcomes were assessed at baseline (month 0), at the end of the intervention (month 3), and at 6 and 12 months follow-up after the end of the intervention (month 9 and 15, respectively). Three assistant researchers, blinded to group allocation, obtained self-reported outcomes using telephone interviews or face-to-face meetings.

### Intervention (IG)

The intervention was conducted in a primary care facility, and all participants reported to the training facility twice a week for 3 months (24 sessions), with no cost to themselves. Each session lasted 60 minutes, and all protocols incorporated the overload training principle [25]. As part of the intervention but without being registered, the physical activity specialist encouraged all subjects to perform a moderate-intensity physical activity such as brisk walking during the days with no program session. The program monitor also tried to find a leader within the group to organize the meetings. All training sessions began with a warm-up (walking at their usual pace for 10 minutes), and ended with a cool-down (stretching for 5 minutes). Standardized sessions were always performed under the supervision of the same physical activity specialist, previously trained and blinded to the study objectives. All sessions included 20 to 30 minutes of an aerobic activity, such as walking at a fast pace. Each session also included upper and lower body strength-based exercises such as rising from a chair, stair climbing, knee bends, floor transfers, lunges, leg squat, leg extension, leg flexion, calf raise, abdominal curl, carrying objects, throwing and catching a ball, and push-ups against the wall. An 8-repetition maximum was established at the first training session, and was repeated at the second training session. Participants were instructed to perform strength training at a perceived exertion intensity of 12–14 (fairly hard) [26], without holding their breath during exercises to minimize exercise-induced blood pressure elevations. The participants initially performed one to two sets of six to eight repetitions of each exercise; the number of repetitions was increased when a participant was able to complete 8 repetitions at a lower perceived exertion intensity; the maximum number of repetitions were 15. This protocol was developed in a pilot training study [27].

During the last two sessions, visits were made to the nearest community resources (e.g. sport facilities), and physical activity professionals were introduced in order to help the participants to continue with the regular physical activity practice. Participants from the program were offered a special monthly rate.

### Control Group (CG)

Subjects who were randomly assigned to the control group were asked to continue their routine daily activities and received their usual care from their primary care practice whenever it was needed. The control subjects were called once a month for social talks with the health professionals.

### Statistical Analyses

All analyses were performed according to the intention-to-treat principle. The primary outcome data was obtained at baseline and at 15-month follow-up for all participants who were randomized (IG = 183; CG = 179). For the secondary outcome measure, all the participants who were randomized were analyzed; some participants who were lost during a previous follow-up were contacted during the next follow-up and assigned the previous value obtained (last observation carried forward). The analysis performed for our secondary outcomes was an intention-to-treat analysis replacing the missing values with the scores obtained in the previous assessment.

Our primary outcome measure was the number of visits at month 15 and the variation of visits in that time (variation of visits before-after) (discrete variable). A Mann Whitney U test (non-parametric statistical test) was performed to assess any difference between groups. A subgroup analysis was performed assessing the effect of the intervention in the subjects with more than 20 visits against the subjects with less than 20 visits at baseline.

An analysis of mixed linear modeling was performed for the secondary outcome measures (self-reported physical function, physical composite score and mental composite score). Mixed linear modeling can be applied to repeated measures data from unbalanced designs [28]. Bonferroni post-hoc tests were used with significant interactions (*P*<.001).

All investigators involved in the data analysis were blinded to the treatment assignment. For the statistical analyses, SPSS version 18.0 software (SPSS, Chicago, IL) was used, and an alpha level of.001 was selected.

## Results

### Individual Characteristics and Compliance with the Protocol

Three hundred and sixty-two individuals were randomized: 183 to the physical activity program (IG) and 179 to the control group (CG). Participants in the IG were required to complete 24 sessions during 3 months, and their compliance was 83%. Compliance was assessed by recording the attendance of each participant at each session (a delay of more than 15 minutes was recorded as non-attendance). There was a dropout rate of 14.8% at month 3 (27 participants did not complete the intervention, and 32 did not attend the last session assessment) (see Figure 1 for more details). There were no adverse events during the study period. At month 9, 161 subjects from the IG and 143 from the CG were assessed. At month 15, 156 subjects from the IG and 158 subjects from the CG were assessed (see Figure 1 for more details). The total dropout rate at month 15 was 23 subjects (12.6%) in the IG, and 21 subjects in the CG (11.7%).

The month 0 (baseline) characteristics of the participants are presented in Table 1.

**Table 1:** Month 0 (baseline) characteristics of intervention and control groups.

Variable	IG (n = 183)	CG (n = 179)
Age (years), mean (SD)	68.3 (8.8)	67.2 (9.1)
Female, number (%)	128 (69.9)	116 (64.8)
Anthropometrics:		
Height (cm), mean (SD)	158.4 (9.7)	160.6 (9.2)
Weight (kg), mean (SD)	69.5 (14.8)	70.9 (13.3)
BMI (kg/m^2^), mean (SD)	28.4 (4.3)	29.6 (4.9)
Marital Status:		
Married, partner alive, number (%)	128 (69.9)	127 (70.9)
Single, never married, number (%)	19 (10.4)	13 (7.3)
Widowed, number (%)	29 (15.8)	34 (19)
Divorced, number (%)	7 (3.8)	5 (2.8)
Medical conditions:		
Hypertension, number (%)	107 (58.5)	102 (57)
Diabetes mellitus, number (%)	49 (26.8)	47 (26.3)
Hypercholesterolemia, number (%)	72 (39.3)	77 (43)
Myocardial infarction, number (%)	21 (11.5)	19 (10.6)
Congestive heart failure, number (%)	15 (8.2)	13 (7.3)
Osteoarticular chronic problems, number (%)	84 (45.9)	91 (50.8)
Number of chronic medications, median (IR)	5 (12)	6 (7)
Baseline number of consultations, mean (SD)	18.2 (7.4)	17.6 (9.7)
Baseline MET – minutes/week, mean (SD)	1186.9(1789.1)	943.6(1917.6)

SD = standard deviation; IR = interquartile range; MET = metabolic equivalent.

### Primary Outcome Measure

As no gender effect was evident in the main outcome data, the data was pooled. The IG and the CG participants had a baseline mean (SD) number of visits/year of 18.2 (7.4) and 17.6 (9.7), respectively. At month 15, the IG had a significantly reduced the number of visits to 14.8 (8.5), and the CG remained with similar data 18.2 (11.1) (*P* = .002). The IG had a greater reduction in the total number of consultations/year to the PHC, when comparing the twelve months prior to (month 0) and after the program (month 15). The effects of the exercise program on the primary outcome measure are shown in Table 2.

**Table 2:** Effects of the exercise program on the total number of visits and variation of visits (before-after) at month 15.

Variable		CG (n = 179)		IG (n = 183)		p-value
Number of consultations (all PHC)	Month 15, mean (SD), (CI 95%)	18.2 (11.1)	(16.5,19.9)	14.8 (8.5)	(13.4,16.2)	–
	Month 15, median (IR), (P25,P75)	15 (11)	(11,22)	14 (10)	(9,19)	.002
	V at month 15, mean (SD)	0.6 (7.7)		−3.4 (7.3)		<.001
Subjects with >20 visits (n = 109)	Month 15, mean (SD), (CI 95%)	27.4 (13.9)	(23.5,31.2)	19.4 (8.7)	(17.1,21.7)	–
	Month 15, median (IR), (P25,P75)	25.5 (19)	(17.3,36)	18 (12)	(13,24.5)	.009
	V at month 15, mean (SD)	−1.7 (10.4)		−6.3 (8.4)		.002
Subjects with ≤20 visits (n = 207)	Month 15, mean (SD), (CI 95%)	14 (6.1)	(12.8,15.1)	12.0 (7.1)	(10.5,13.4)	–
	Month 15, median (IR), (P25,P75)	13 (8)	(10,18)	10 (9)	(7,16.3)	<.001
	V at month 15, mean (SD)	1.6 (5.8)		−1.7 (6)		<.001
Subjects that reduced the numberof visits at month 15:						
All PHC	N (%)	85 (47.5)		134 (73.2)		<.001*
Subjects with >20 visits (n = 125)	N (%)	37 (61.7)		55 (84.6)		.007*
Subjects with ≤20 visits (n = 237)	N (%)	53 (41.1)		72 (66.7)		<.001*

Note: Mann Whitney U test was performed unless indicated.

* Chi Square test was performed.

IG = Intervention group; CG = Control group; V = variation of visits (before-after); SD = standard deviation; IR = interquartile range; CI = confidence interval; PHC = Primary Healthcare Centers.

### Secondary Outcome Measures

A significant (*P*<.001) group effect was observed for all month 3, 9 and 15 measures; the IG performed better than the CG for every dependent variable. No gender effect was identified, so the data were pooled. The effects of the exercise program on selected secondary outcome measurements are shown in Table 3.

**Table 3:** Measures of self-reported health status.

Variable	Interval	IG (n = 183)		CG (n = 179)		p-value*	Effect tested	Contrasts**
		Mean (SD)	95% CI	Mean (SD)	95% CI			
SF-12 (0–100)								
PF (points)	Month 0	38.8 (8.5)	(32.2,40.9)	39 (9.1)	(32.6,42)	<.001	Group*Time	Month 0-month 3; p<.001
	Month 3	43.6 (5)	(40.6,46.1)	38.4 (7.2)	(29.1,42.8)			Month 0-month 9; p = .001
	Month 9	41.0 (7.2)	(37.8,43.6)	38.3 (8)	(29,41.7)			Month 0-month 15; p = .047
	Month 15	38.2 (8.8)	(33.8,43.4)	37.4 (7.4)	(30,39.4)			Month 3-month 15; p = .062
PCS (points)	Month 0	41.8 (7.6)	(36.1,46.2)	40.5 (7.7)	(35.3,45.2)	<.001	Group*Time	Month 0-month 3; p<.001
	Month 3	46.4 (8.6)	(41.1,49.5)	38.6 (4.6)	(33.3,42.8)			Month 0-month 9; p<.001
	Month 9	44.3 (6.4)	(39.3,47.1)	39.3 (6.2)	(29.5,41)			Month 0-month 15; p = .030
	Month 15	45.4 (6.4)	(41.6,48.4)	38.7 (9.8)	(27.4,40.3)			Month 3-month 15; p = .001
MCS (points)	Month 0	34.6 (7.4)	(30.3,41.2)	35.2 (6.4)	(31.6,40.6)	<.001	Group*Time	Month 0-month 3; p<.001
	Month 3	41.3 (5.3)	(33.5,45.6)	32 (4.2)	(29.3,36.3)			Month 0-month 9; p = .001
	Month 9	39.3 (8)	(29.4,37)	31.2 (5.5)	(26.8,35.1)			Month 0-month 15; p = .011
	Month 15	38.9 (6.4)	(34.6,42.2)	30.8 (7.1)	(25.2,36.7)			Month 3-month 15; p = .026

*Note:* Means and standard deviations are reported for each outcome measure at month 0 and at month 3, 9 and 15 until completion of the study. Means were generated using participants with data at least three time points for the outcome of interest. P-values are based on linear mixed modelling. An increase in the scores of the SF-12 scales means an improvement in the perceived rate of wellbeing.

* P-values are interpreted from the results of comparisons between specific time points. When the p-value interpreted is from the group-by-time interaction effect, the change between two time points for the two groups is compared.

** Bonferroni post-hoc tests were used with significant interactions in the IG (p<.001).

IG = intervention group; CG = control group; PF = physical function; PCS = physical composite score; MCS = mental composite score; SD = standard deviation; CI = confidence interval.

According to the self-reported physical function, physical composite score and mental composite score, documented via the SF-12 version 2 survey, the IG participants showed significant greater improvements than those in the CG from month 0 (baseline) to month 3 measures (end of training) (*P*<.001), that were sustained in the month 9 follow-up testing, with significant group-by-time interactions by the end of the study (see Table 3). The physical composite score and the mental composite score measures were also sustained in the month 15 follow-up in the IG (*P* = .001, *P* = .026, respectively from month 3 to month 15); however, detraining induced decreases in physical function measures in the IG participants (*P* = .062, from month 3 to month 15).

## Discussion

The three major findings of this study were that: (1) a standardized physical activity program linked to community resources was effective in decreasing the total number of visits to the PHC in inactive patients, (2) the program induced improvements in self-reported quality of life, and (3) these improvements were sustained 12 months after the end of the training program.

Our physical activity program was effective in decreasing PHC use in terms of the total number of consultations per year. Aside from primary health benefits from increased physical activity, such as a longer term reduction in the incidence or severity of clinical disease [29], health-related quality of life benefits may be more immediate and, for at least the patients who continued with a regular physical activity practice, substantial through a decrease number of medical appointments. Enhanced well-being among previously inactive individuals not only would help sustain continued physical activity but is itself an outcome that patients’ value, seek health care for, and naturally use to appraise the benefit of their treatments [30].

Effective promotion of exercise could result in substantial healthcare savings in terms of health improvements and decreases in health system use. However, physical activity promotion interventions should be aimed at achieving sustained increases in physical activity. Community-based programs have an advantage over hospital programs in their potential to provide continuity of care [14], and to link patients with appropriate alternative locations to perform regular physical activity. In Europe, physical activity is promoted in a variety of ways – for example, exercise referral schemes in primary care [31]. However, the success of referral schemes has yielded conflicting results. Such referrals schemes have not been particularly effective in increasing physical activity beyond 12 weeks (the normal period of support within an intervention), and certainly not over years [31]. GPs recommend exercise for several indications, but access to, and familiarization with some local exercise promotion programs might be necessary. The profile of inactive patients who tend to overuse PHC have greater relationships and trust with PHC professionals than with sport facility workers, so PHC might be an optimal place to familiarize patients with regular physical activity and encourage them to continue with a healthy habit acquired in the PHC itself. Our program included visits to the nearest community resources (e.g. sport facilities) in order to help the patients to continue with regular physical activity practice.

In Catalonia, each medical appointment with a specialist or nurse costs between 21€ and 36€, an emergency visit between 54€ and 88€, and a home visit between 28€ and 58€ [32]. The mean number of consultations per patient at baseline and at month 15 including both the IG and the CG participants, was 17.9 (8.7) and 16.6 (10.1) respectively. The mean number of consultations per patient included in the IG at baseline and at month 15, was 18.2 (7.4) and 14.8 (8.5), respectively; thus the overall cost saving for the number of consultations over the 15-month study was 161.5€ per participant. The total cost of a physical activity specialist for a 24-session program was 480€ (20€/session), and the material needed for the program cost 20€ (two soft-balls and elastic bands). Each group had around 20 participants, so that the cost of the program/participant was around 25€. This means that there would be a saving of 136.5€ per participant, without taking into account the possible savings in medication and other indirect measures (not analyzed in the present article). Similarly, the World Health Organization [33] showed that physical inactivity costs between 150€ and 300€ per person/year in European countries, and Nelson et al. [34] showed that physical activity could reduce health costs after one year of its practice. Our training program increased the number of METs in the IG from baseline, which were sustained 12 months after the intervention: IG = 1477.7 (1019.1); CG = 1142.6 (1595.9) (data in publication process).

Substantial evidence documents the health benefits of regular physical activity [35]. Many of the beneficial effects of physical activity are particularly salient for mid-life and older adult populations [36], [37]. We could speculate that the subjects who continued with a regular physical activity practice mostly reduced the number of visits. The most important approach in physical activity promotion strategies should focus on adherence offering different adequate choices to help the patients to continue with the regular physical activity practice.

The stimulus of the program induced improvements in self-reported physical function, physical and mental composite score at month 3. Previous studies had shown that increases in physical activity levels had yielded improvements in the symptoms of depression [38]. They also showed that walking had a statistically significant, large effect in some populations, although the current evidence base from randomized, controlled trials is limited [38].

In another systematic review on exercise showed a moderate to large positive impact in quality of life of depressed individuals, especially in components related to physical and psychological domains [39]. An improvement in quality of life has been linked to an improvement in physical function [40]. There is evidence in the literature to suggest that measures of physical function in adults were also related to feelings of well-being [41]. Our program had beneficial effects on self-reported health outcomes, which might also be linked to decreased rates of PHC use.

The improvements in the physical composite score and the mental composite score in the IG were sustained 12 months after the training ended (month 15). A possible mechanism for this sustainability is that an increasing of physical activity levels might have maintained feelings of well-being. The duration and intensity of our training program, as well as its multicomponent nature induced improvements in self-reported physical function; however, detraining induced decreases at month 15. Self-reported physical function assesses the impact of health on the performance of activities ranging from basic self care to vigorous physical activity. At month 15, the patients in the IG group maintained and increased PA levels, mainly with an aerobic activity such as walking, which still improved mental and physical composite score, but wasn’t specific enough to improve physical function. Previous studies have shown the importance of the exercise being task specific if functional ability is to be improved [42], [43]. The duration of training has also been suggested to be an important contributing factor to the retention of neuromuscular adaptations once training has ended [44]. Attenuation or reversal of functional decline in this population is clinically relevant, suggesting that habitual PA based on an aerobic activity is insufficient to maintain physical function. Therefore, physical activity programs should be linked to local sport resources, in order to facilitate the continuity of a functional-based exercise program.

The CG showed no significant changes in their month 15 measures, with respect to their month 0.

There are some limitations to this efficacy trial. The present study only focused on primary care use; future studies should assess whether physical activity programs yield improvements in other direct measures such as number of medications, as well as in other effects on health costs, such as hospital institutionalization. However, GPs play the gatekeeper role in the Catalan Health System, being the first point of contact with the system, except for hospital emergencies. Patients are advised to use primary health care emergency services over hospital emergency wards for non-life-threatening conditions.

The intervention took place in 8 non-random PHC, which could have biased the results and alter generalizability, due to the voluntary centers being more interested in the topic of study. However, the main outcome measure was registered by professionals not involved in the study, and unaware of the group allocation of the patient.

The screening question used to recruit the study sample could have selected a highly active population. However, Hubbard et al. [36] showed that exercise conferred its greatest benefits to improvements in health status to those with the highest number of health deficits at baseline (i.e. the most frail). Thus, differences between groups could have been greater with a less restrictive criterion.

### Conclusions

In summary, our findings indicate that a 3-month physical activity program linked to community resources is a short-duration, effective and sustainable intervention in inactive patients to decrease rates of PHC use and improve self-reported quality of life. It is therefore a potentially suitable program for clinical settings and primary care centers.

## Supporting Information

Checklist S1 **CONSORT Checklist.**
(DOC)

Protocol S1 **Trial Protocol.**
(PDF)
