# Supplementary material for: The Effect of a Physical Activity Program on the Total Number of Primary Care Visits in Inactive Patients: A 15-Month Randomized Controlled Trial
Source: PLoS One. 2013 Jun 21;8(6):e66392. doi: 10.1371/journal.pone.0066392 (PMC3689840; doi:10.1371/journal.pone.0066392)
Supplement: Protocol S1 — Trial Protocol. (PDF) [file pone.0066392.s002.pdf]

ClinicalTrials.gov
A service of the U.S. National Institutes of Health

Trial record **1 of 2** for: Referral from primary care to a physical activity programme:
Previous Study | Return to List | Next Study

Referral From Primary Health Care Centers to a Physical Activity Program. A Randomized Controlled Trial

The recruitment status of this study is unknown because the information has not been verified recently.

Verified July 2008 by Jordi Gol i Gurina Foundation.
Recruitment status was Not yet recruiting

Sponsor:
Jordi Gol i Gurina Foundation

Collaborators:
Unitat de Suport a la Recerca, Barcelona, Spain
Catalan Society of Family Medicine

Information provided by:
Jordi Gol i Gurina Foundation

ClinicalTrials.gov Identifier:
NCT00714831

First received: July 11, 2008
Last updated: NA
Last verified: July 2008
History: No changes posted

Full Text View | Tabular View | No Study Results Posted | Disclaimer | How to Read a Study Record

Purpose

Declining **physical activity** and the associated rising burden of disease is a major public-health problem and it has long been known that being physically **active** has positive health effects. The virtual absence of a public health practice infrastructure for the promotion of **physical activity** at the local level presents a critical challenge to control policy for chronic disease. We aimed to assess the efficacy of a 3-months **physical activity program** in **primary health care** to create adherence to **physical activity** in sedentary individuals.

Condition	Intervention
Sedentary General Population	Behavioral: **Physical activity** intervention<br>Behavioral: Health educational **program**

Study Type: Interventional
Study Design: Allocation: Randomized
Endpoint Classification: Efficacy Study
Intervention Model: Parallel Assignment
Masking: Single Blind (Outcomes Assessor)
Primary Purpose: Prevention

Official Title: Referral From **Primary Health Care** Centers to a **Physical Activity Program:** Establishing Long-Term Adherence? A Randomized Controlled Trial

Further study details as provided by Jordi Gol i Gurina Foundation:

Primary Outcome Measures:

- This randomized controlled trial was designed to assess the effectiveness of a **primary care physical activity** intervention, in increasing adherence to **physical activity** in general population, as well as their self-reported **physical activity**. [Time Frame: baseline-3-6-12-18] [Designated as safety issue: No]

Estimated Enrollment: 424
Study Start Date: September 2008
Estimated Study Completion Date: December 2010
Estimated Primary Completion Date: September 2010 (Final data collection date for primary outcome measure)

Arms	Assigned Interventions
**Active** Comparator: CG<br>Control Group	Behavioral: Health educational **program**<br>Health education sessions and stretching sessions
Experimental: IG<br>Intervention Group	Behavioral: **Physical activity** intervention<br>3-months **physical activity** intervention

Detailed Description:

Physical inactivity has emerged as an important risk factor for many chronic diseases. The decline in physical activity is a key public health concern.

Objective. The objective of the study is to assess the effectiveness of a 3-months physical activity programme in the primary healthcare centres (HC), combined with community resources and interdisciplinary work, to create adherence to physical activity in sedentary individuals.

Methods. The design of the study is a randomized controlled trial with a randomized selection of the sample. A total of 424 subjects of both sexes, older than 18 years old, with a low physical activity level (IPAQ), independent, with no medical contraindication to practice physical activity, and coming from 8 different HC, will participate in the study. Each subject will be offered to participate voluntarily in a physical activity programme (24 sessions, twice a week, 60 minutes/session). There will be two groups of 15-20 subjects in each HC (a total of 8 HC), randomizing the subjects of the intervention (GI) and control groups (GC). The study will be divided in two phases, and in each phase there will be 16 groups (8 GC and 8 GI).

In the first and last session different parameters will be assessed in all the subjects (GC and GI): (1) quality of life related to health with SF-12 questionnaire, (2) the attitude towards practicing physical activity with Prochaska stage changes, (3) the physical activity level with the reduced version of the IPAQ, (4) health perception with COOP/WONCA questionnaire, and (5) the social support for physical activity with SSPAS. The welfare pressure to the HC of each participant is also registered, during the 6 months previous and after the programme. Subjects of GI will go through the 24 sessions while subjects of GC will go through a health educational program and will be asked to continue with their everyday activities. After 3, 6 and 12 months of the end of the programme, a pursuit by means of a call will be done (IPAQ, SF-12 and SSPAS). After 6 and 12 months of the end of the programme the same call will be used to assess the Prochaska stages.

Eligibility

Ages Eligible for Study:	18 Years and older
Genders Eligible for Study:	Both
Accepts Healthy Volunteers:	No

Criteria

Inclusion Criteria:

- Adults of both gender.
- Low physical activity level assessed with International Physical Activity Questionnaire.
- Able to walk and stand up from a chair unaided.

Exclusion Criteria:

- Recommended contradictions to advising physical activity to older adults.
- Presence of unstable angina.
- Uncontrolled congestive heart failure.
- Unstable arrhythmia or heart valvular disease.
- Progressive or debilitating medical conditions.
- Severe hypertension (systolic ≥ 200, or diastolic ≥ 120).

Contacts and Locations

Please refer to this study by its ClinicalTrials.gov identifier: NCT00714831

Contacts

Contact: Maria Giné-Garriga, MS   +34 675785983   mgine.pbcn@ics.scs.es

Contact: Carme Martin-Borràs, MS   +34 932594338   cmartinb.bcn.ics@gencat.cat

Locations

Spain

Primary Health Care   **Not yet recruiting**

Barcelona, Spain, 08022
Contact: Maria Giné-Garriga, MS   +34 675785983   mgine.pbcn@ics.scs.es
Principal Investigator: Maria Giné-Garriga, MS
Principal Investigator: Carme Martin-Borràs, MS
Sub-Investigator: Carlos Martin, PhD, MD
Sub-Investigator: Anna Puig-Ribera, PhD
Sub-Investigator: Juan José Anton, MD
Sub-Investigator: Agustí Guiu, MD
Sub-Investigator: Ana Cascos, Nurse

Sponsors and Collaborators

Jordi Gol i Gurina Foundation

Unitat de Suport a la Recerca, Barcelona, Spain

Catalan Society of Family Medicine

Investigators

Study Director: Carlos Martin, PhD, MD   **Primary Health Care** of Barcelona

More Information

No publications provided by Jordi Gol i Gurina Foundation

Additional publications automatically indexed to this study by ClinicalTrials.gov Identifier (NCT Number):

Giné-Garriga M, Martin C, Martín C, Puig-Ribera A, Antón JJ, Guiu A, Cascos A, Ramos R. Referral from primary care to a physical activity programme: establishing long-term adherence? A randomized controlled trial. Rationale and study design. BMC Public Health. 2009 Jan 22;9:31.

Responsible Party:	Maria Giné-Garriga, Primary Health Care of Barcelona
ClinicalTrials.gov Identifier:	NCT00714831 History of Changes
Other Study ID Numbers:	PI081304
Study First Received:	July 11, 2008
Last Updated:	July 11, 2008
Health Authority:	Spain: Comité Ético de Investigación Clínica

Keywords provided by Jordi Gol i Gurina Foundation:
Physical activity promotion
Primary health care
Adherence

ClinicalTrials.gov processed this record on November 25, 2012
